# Enhanced Anti-Inflammatory Effects of Rosemary (*Salvia rosmarinus*) Extracts Modified with *Pseudomonas shirazensis* Nanoparticles

**DOI:** 10.3390/antiox14080931

**Published:** 2025-07-29

**Authors:** Enrique Gutierrez-Albanchez, Elena Fuente-González, Svitlana Plokhovska, Francisco Javier Gutierrez-Mañero, Beatriz Ramos-Solano

**Affiliations:** 1 Faculty of Pharmacy, Universidad San Pablo-CEU Universities, 28040 Madrid, Spain; enrique.gutierrezalbanchez@ceu.es (E.G.-A.); e.fuente@usp.ceu.es (E.F.-G.); svetaplohovska@gmail.com (S.P.); jgutierrez.fcex@ceu.es (F.J.G.-M.); 2 Institute of Food Biotechnology and Genomics, NAS of Ukraine, 01112 Kyiv, Ukraine

**Keywords:** *Salvia rosmarinus*, silver nanoparticles, secondary metabolism, beneficial bacteria

## Abstract

Rosemary (*Salvia rosmarinus*) is renowned for its antioxidant, anti-inflammatory, and antihyperglycemic properties, largely attributed to its rich phytochemical profile. This study evaluates the potential of metabolites from *Pseudomonas shirazensis* NFV3, formulated in silver nanoparticles (AgNPs), to enhance the bioactivity of rosemary extracts in postharvest applications. Rosemary stems were treated with AgNPs coated with bacterial metabolites (NP), bacterial cells, or metabolites (LM), and the extracts’ phytochemical composition and bioactivities were assessed. HPLC and HPLC–MS analyses revealed that the NP treatment induced significant metabolic remodeling, particularly upregulating rosmarinic acid and selected triterpenes (ursolic and betulinic acids), while reducing carnosic acid levels. NP-treated extracts exhibited significantly enhanced inhibition of cyclooxygenase (COX-1 and COX-2), indicating improved anti-inflammatory potential. The α-glucosidase inhibition and antioxidant activity (DPPH assay) of the extracts were not substantially altered, suggesting the selective enhancement of pharmacological functions. These findings demonstrate that nanoparticle-based elicitation selectively remodels secondary metabolism in rosemary, improving extract quality and bioactivity. This strategy offers a novel, sustainable tool for optimizing plant-based therapeutics in the phytopharmaceutical industry.

## 1. Introduction

*Salvia rosmarinus* (syn. *Rosmarinus officinals* L.) [1], commonly known as rosemary, is a perennial, aromatic shrub, belonging to the *Lamiaceae* family, and native to the Mediterranean region. Traditionally cultivated for both culinary and medicinal purposes, rosemary has been integrated into the daily lives of Mediterranean populations for centuries. Its historical applications span a variety of ailments, including digestive complaints, headaches, muscle pain, and memory enhancement, reflecting its broad therapeutic reputation in folk medicine [2,3]. The growing demand for natural, plant-based therapeutics has propelled rosemary extract-based products to the forefront of the nutraceutical and functional food markets. The global rosemary extract market was valued at approximately USD 260 million in 2024 and is projected to reach USD 377 million by 2032, with a compound annual growth rate (CAGR) of 5.9% [4]. This expansion is fueled by consumer preferences for clean-label, natural ingredients and the recognition of rosemary’s antioxidant and anti-inflammatory benefits across food, pharmaceutical, and personal care sectors.

The phytochemical richness of rosemary is central to its pharmacological potential. Its leaves are abundant in polyphenols, flavonoids, and terpenoids, with the presence of the phenolic diterpenes, carnosic acid and carnosol, standing out, as well as rosmarinic acid. These compounds are recognized for their potent antioxidant and anti-inflammatory properties, which are believed to underlie many of rosemary’s health-promoting effects [5,6,7]. In addition, rosemary contains other bioactive constituents, such as triterpenes (e.g., ursolic acid, betulinic acid), phenolic acids, and volatile terpenoids (e.g., 1,8-cineole, α-pinene), which are major components of its essential oil fraction, further contributing to its therapeutic versatility [8]. Rosemary’s role in the Mediterranean diet is not only culinary, but also therapeutic, as its regular consumption has been associated with reduced risk of chronic diseases that are prevalent in Mediterranean populations [9,10]. Pharmacological surveys have documented its use for metabolic, inflammatory, and neurocognitive disorders, supporting the scientific investigation of the traditional health claims related to its use [11,12].

Contemporary research has elucidated several mechanisms underlying the health-promoting properties of rosemary extracts, with particular emphasis on their anti-inflammatory, antihyperglycemic, and antioxidant effects. The anti-inflammatory activity of rosemary is primarily attributed to the modulation of key molecular pathways, including the inhibition of nuclear factor kappa B (NF-κB) activation, the downregulation of pro-inflammatory cytokines (e.g., TNF-α, IL-1β, IL-6), and the suppression of cyclooxygenase-2 (COX-2) and inducible nitric oxide synthase (iNOS) expression [5,13]. Notably, both carnosic acid and carnosol have demonstrated the capacity to attenuate inflammatory responses in vitro and in vivo, with evidence suggesting that whole rosemary extracts may exert synergistic effects beyond those of isolated compounds [7,14].

The antihyperglycemic properties of rosemary have been substantiated in several preclinical models of type 2 diabetes mellitus, wherein the administration of rosemary extracts or their polyphenolic constituents resulted in significant reductions in fasting plasma glucose, improved lipid profiles, and enhanced antioxidant defense systems. Mechanistically, these effects are linked to the inhibition of intestinal glucosidase enzymes, the enhancement of insulin sensitivity, and the upregulation of endogenous antioxidant enzymes [6,10]. Recent human studies, though limited, have indicated that rosemary tea consumption can lead to significant improvements in glycemic control among individuals with type 2 diabetes, supporting its potential use as a complementary metabolic regulator [9].

Rosemary’s antioxidant capacity is largely ascribed to its high content of phenolic diterpenes and polyphenols, which act as free radical scavengers, metal ion chelators, and modulators of redox-sensitive signaling pathways, such as Nrf2 [15]. In vitro and in vivo studies have consistently demonstrated that rosemary extracts can inhibit lipid peroxidation, reduce oxidative stress markers, and enhance the activity of endogenous antioxidant enzymes, such as superoxide dismutase (SOD) and glutathione peroxidase (GPx) [5,10,16]. These findings have prompted the widespread application of rosemary extracts as natural antioxidants in the food industry and as potential therapeutic agents for oxidative stress-related disorders.

These pharmacologically active molecules are the plant’s secondary metabolites, whose role is to aid the plant’s adaptation to changing environmental conditions, and, therefore, they are inducible. Among the strategies to trigger plant secondary metabolism, beneficial bacterial strains and their metabolites have been used successfully [17,18]. Recent advances in nanotechnology have opened up new avenues for enhancing plant metabolism by formulating bacterial metabolites in nanoparticles, i.e., the metabolites are used as bioreductants of the metals, generating a nanoparticle coated with bacterial metabolites that have biological activity [19]. The biological synthesis of nanoparticles using bacterial metabolites or plant extracts (green synthesis) offers several advantages, including biocompatibility, reduced toxicity, and environmental sustainability, aligning with the growing demand for natural and safe therapeutic products [20,21]. A specific type of nanoparticles obtained by green synthesis was found to improve key phytochemical contents of rosemary, potentially amplifying their anti-inflammatory, antihyperglycemic, and antioxidant effects [22,23]. Bacterial metabolites act as reducing and capping agents during the synthesis of metallic or polymeric nanoparticles, enabling the development of hybrid systems that harness both the therapeutic potential of rosemary and the functional benefits of nanotechnology [24]. Preliminary in vitro and in vivo studies indicate that bacterial metabolite-loaded nanoparticles exhibit enhanced cellular uptake, with a consequent higher level of phytochemical modulation of the plant extract [20,23]. Unlike previous studies employing either silver nanoparticles or generic bacterial metabolites, this work uniquely demonstrates the selective remodeling of rosemary’s secondary metabolism through the postharvest application of AgNPs coated with metabolites from *Pseudomonas shirazensis*, a strain not previously reported as being used for this purpose.

Among the beneficial strains, the genus *Pseudomonas* has a number of strains that are able to trigger plant metabolism [25]. Strain *Pseudomonas shirazensis* NFV3 has shown potential to stimulate the plant immune system [18], so we reasoned that it could stimulate rosemary metabolism, increasing the phytochemical concentration and, therefore, its antioxidant potential and health benefits. To reach this objective, the strain, its metabolites, and a formulation of these metabolites in silver nanoparticles were delivered to rosemary stems and, after metabolomic characterization, its ability to inhibit alfa-glucosidase, as an indicator of antihyperglycemic activity, and COX-1 and 2, as indicators of anti-inflammatory potential, were evaluated.

## 2. Materials and Methods

### 2.1. Biological Synthesis of Nanoparticles

Silver nanoparticles (AgNPs) were synthesized following the protocol described by Plokhovska [24]. Briefly, a 24 h culture of *Pseudomonas shirazensis* NFV3 (CECT 31128) was centrifuged on a (AFI LISA MultiLab Centrifuge, France), and the supernatant was filtered through a 0.25 µm membrane (VWR International, Radnor, PA, USA) to remove bacterial cells. The resulting filtrate was mixed with 1 mM AgNO_3_ at a ratio of 2:4 (*v*/*v*) and incubated at 37 °C for 24 h. The synthesized AgNPs were characterized by UV–Vis spectroscopy (λ = 430 nm) on a SPECTROstar Nano spectrometer (BMG LABTECH, Ortenberg, Germany) and transmission electron microscopy (TEM a Thermo Fisher Scientific Prisma E and xT Microscope Control v16.2.2 software), revealing the occurrence of spherical nanoparticles with an average diameter of 20.71 ± 0.43 nm and an organic corona composed of bacterial metabolites. The AgNPs were lyophilized and resuspended in Milli-Q water to a stock concentration of 6000 µg/mL; a working solution of 60 µg/mL was prepared for the experiments (NP). The bacterial filtrate (ML) and corresponding cells were also used as treatments (LM, cells, respectively).

The datasets generated and/or analyzed during the current study are available from the Zenodo repository (https://doi.org/10.5281/zenodo.14051744).

### 2.2. Plant Material and Experimental Setup

A total of twelve (*Salvia rosmarinus*) plants, cultivated under homogeneous conditions, were selected for this study. From each plant, apical stem segments, measuring 15 cm in length, were randomly excised to ensure sample homogeneity.

Four different treatments were prepared, as a follows:

1. Control;
2. Cells (bacterial cells suspended in water);
3. LM (likely a liquid medium containing bacterial metabolites);
4. NP (bacterial metabolite LM formulated in silver nanoparticles).

Each treatment was applied to three independent rosemary branches (*n* = 3 per treatment). For the application, 5 mL of the corresponding solution was uniformly sprayed over the surface of each branch, using a fine-mist sprayer. An additional set of three untreated branches was collected to serve as the negative control group.

All the branches, treated and untreated, were air dried under controlled laboratory conditions until they reached a constant weight, ensuring complete desiccation.

### 2.3. Sample Processing and Extraction Procedure

After drying, each rosemary branch (previously treated and used as a replicate) was processed individually. The leaves were carefully separated from the stems and ground using a mortar and pestle in the presence of liquid nitrogen to facilitate sample pulverization.

The extraction protocol using 75% ethanol and overnight maceration was adapted from previous optimization studies involving rosemary [26,27], which demonstrated maximum polyphenol and flavonoid recovery under these conditions. To extract the bioactive compounds, 0.5 g of the powdered material from each replicate was subjected to maceration in 10 mL of 75% ethanol (*v*/*v*). The extraction was performed overnight at room temperature (22 °C) in dark conditions to minimize the photodegradation of sensitive metabolites. After maceration, the suspensions were centrifuged at 1100 rpm for 20 min. The supernatant was collected, and the pellet was discarded. This supernatant was then filtered through a 0.2 μm cellulose acetate membrane to remove particulate matter. The resulting filtrate was concentrated using a rotary evaporator (R210-Buchi Labortechnik AG, Flawil, Switzerland). until complete removal of the ethanol–water solvent phase was achieved.

The resulting dry extract was subsequently resuspended in the original volume (10 mL) of 100% ethanol to ensure concentration consistency across the replicates. A final filtration through a 0.2 μm cellulose acetate filter was performed to ensure extract purity. The extracts were stored at −20 °C in amber, airtight glass vials to protect them from light and oxidation until further use.

### 2.4. Quantification of Active Compounds Using HPLC

The chemical composition of the ethanolic extracts was analyzed using a modified version of the protocol described by Wellwood & Cole [26]. The analysis was carried out using an Agilent 1100 Series HPLC system, equipped with a G1315A diode array detector (DAD). Chromatographic separation was achieved using an Inertsil ODS-3V column (4.6 × 150 mm, 5 μm particle size, 100 Å pore size; GL Sciences, Eindoven, The Netherlands). The mobile phase consisted of the following:

1. Phase A: 840 mL Milli-Q water + 82.5 mL acetic acid + 150 mL acetonitrile;
2. Phase B: 100% methanol.

The gradient started at 90% of mobile phase A, gradually shifting to 0% over 30 min. Following the gradient, the column was re-equilibrated with 90% of mobile phase A for 10 min to ensure reproducibility and retention time stability across the injections. The flow rate was set at 1.5 mL/min, and the column temperature was maintained at 40 °C.

Detection was performed at 284 nm, with a ±30 nm bandwidth, while the full UV absorption spectra were recorded from 200 to 800 nm for each peak to support compound identification. Active constituents were identified based on the retention times and spectral profiles, compared to authentic standards, and quantified using calibration curves, constructed from standard compounds.

Quantification was based on external calibration curves prepared using authentic standards (rosmarinic acid (Ref.536954), carnosic acid (Ref. PHR2208), and carnosol (Ref. C9617), Sigma Aldrich, Merck KGaA, Darmstadt, Germany). The linearity (R^2^ > 0.995) of each curve is presented in Supplementary Figure S1.

### 2.5. Metabolic Profiling Using HPLC–MS Scan Mode

The metabolic profiling of *Salvia rosmarinus* extracts was carried out using high-performance liquid chromatography coupled to mass spectrometry (HPLC–MS). The analyses were conducted at the Structural Molecular Analysis Unit of the SIDI (Interdepartmental Research Service, Autonomous University of Madrid) (https://www.uam.es/uam/sidi/unidades-de-analisis/unidad-analisis-elemental-quimico-isotopico/cromatografia, accessed on 1 May 2025), following the protocol proposed by Sharma et al. [27]. In summary, chromatographic separation was performed using a Bruker UHPLC/MS Triple Quadrupole ELITE system, equipped with an HPG1300 binary pump, automatic degasser, column oven (maintained at 40 °C), and a thermostatted autosampler BR840, set at 4 °C. The mobile phase consisted of solvent A (Milli-Q water with 0.1% formic acid) and solvent B (acetonitrile with 0.1% formic acid), delivered at a constant flow rate of 0.5 mL/min. The following gradient program was applied: 0.00 min, 95% A/5% B; 6.00 min, 85% A/15% B; 21.00 min, 75% A/25% B; 23.00 min, 50% A/50% B; 24.10 min, 0% A/100% B; 28.10 min, 95% A/5% B; ending at 32.00 min with the initial conditions. Mass spectrometric detection was carried out using an ELITE triple quadrupole analyzer, equipped with HESI (Heated Electrospray Ionization) and APCI sources. For the HESI in negative ion mode, the following parameters were set: a spray voltage of 5000 V, a capillary (cone) temperature of 350 °C, a cone gas flow of 40 units, a heated probe temperature of 400 °C, a probe gas flow of 50 units, a nebulizer gas flow of 60 units, and the exhaust gas system was activated. The identification of compounds was conducted according to the respective retention times, as in [27].

### 2.6. Total Phenolic Content (TPC)

The total phenolic content was determined spectrophotometrically, using a Biomate-5 UV–visible spectrophotometer (a SPECTROstar Nano spectrometer (BMG LABTECH, Germany)). A volume of 50 μL of the previously prepared ethanolic extract was used for each analysis. The Folin–Ciocalteu method, adapted from Benvenuti [28] with slight modifications, was employed. Briefly, Folin–Ciocalteu reagent (Sigma Aldrich, Merck KGaA, Darmstadt, Germany) was added to the extract, followed by sodium carbonate (Na_2_CO_3_). After 30 min of incubation in the dark at room temperature, the absorbance was measured at 765 nm. The results were expressed as milligrams of gallic acid equivalent per gram of dry extract (mg GAE/g), based on a standard calibration curve of gallic acid.

### 2.7. Total Flavonol Content (TFC)

The total flavonol content was quantified according to the colorimetric method proposed by Zhishen [29], with modifications. For each sample, 1 mL of the ethanolic extract was reacted with aluminum chloride (AlCl_3_) and sodium acetate under controlled conditions. Absorbance was measured at 510 nm, and quantification was based on a calibration curve prepared using catechin. The results were expressed as milligrams of catechin equivalent per gram of dry extract (mg CE/g).

### 2.8. Antioxidant Capacity (AC) Using DPPH Assay

The antioxidant capacity (TAC) of the extracts was assessed using the colorimetric DPPH assay described by Di Sotto [30], with some modifications, using 0.11 mM of DPPH solution and measuring the decrease in absorbance at 517 nm and using gallic acid as a positive control. The assay was performed in duplicate, using three replicates per sample. Gallic acid was used as positive control and the IC_50_ (g/mL) was calculated for each extract.

### 2.9. Inhibition of α-Glucosidase Activity

The inhibitory activity of the extracts on α-glucosidase was evaluated using a microplate-based assay. The assay utilizes the α-glucosidase enzyme from *Saccharomyces cerevisiae* (Sigma Aldrich, G5003-1KU, Merck KGaA, Darmstadt, Germany) and 4-nitrophenyl α-D-glucopyranoside (PNPG, Sigma Aldrich, N1377-1G, Merck KGaA, Darmstadt, Germany) as the substrate. Phosphate buffer (0.1 M, pH 7.5) was prepared using 0.144 g of KH_2_PO_4_, 9 g of NaCl, and 0.795 g of Na_2_HPO_4_·7H_2_O per liter of distilled water.

Test samples were dissolved in dimethyl sulfoxide (DMSO). The enzyme solution was prepared at a concentration of 0.2 U/mL by dissolving 1 mg of α-glucosidase in 50 mL of Milli-Q water, which was diluted to a final concentration of 0.015 U/mL per well. The PNPG substrate was freshly prepared, with a concentration of 4 mM, and diluted to a final concentration of 1 mM in each well, with a total reaction volume of 200 μL.

All the reagents and equipment were preheated to 37 °C prior to use. In each well of a 96-well microplate, 125 μL of phosphate buffer, 10 μL of sample solution, 15 μL of enzyme solution, and 50 μL of PNPG substrate were added, sequentially. The reaction mixture was incubated at 37 °C, and the formation of p-nitrophenol was monitored by measuring the absorbance at 405 nm every 30 s for 35 min, using a microplate reader. The percentage inhibition of α-glucosidase activity was calculated for each sample concentration using the formula: % Inhibition = (Blank slope − Sample slope)/Blank slope) × 100, where the blank refers to wells without inhibitor. The IC_50_ value was determined from the plot of the sample concentration versus the percentage inhibition. All the measurements were performed in triplicate, and fresh substrate was prepared on the day of the experiment to ensure optimal results.

### 2.10. Inhibition of Cyclooxygenase Activity (COX)

Cyclooxygenase (COX) activity was assessed using the Cayman Chemical COX Activity Assay Kit (Item No. 760151), following the manufacturer’s instructions, with minor adaptations as required. This colorimetric assay quantifies the peroxidase activity by monitoring the oxidation of N, N, N’,N’-tetramethyl-p-phenylenediamine (TMPD), which results in a measurable color change at 590 nm.

All the reagents were equilibrated to room temperature before use. The assay was performed using a 96-well plate, with a final volume of 210 µL per well. Each sample was analyzed in triplicate, alongside background and standard controls. For the background determination, 150 µL of each sample was boiled for five minutes, centrifuged, and the supernatant was used as the background control. The plate was set up as follows: COX standard wells received 150 µL of assay buffer, 10 µL of hemin, and 10 µL of COX standard; background wells received 120 µL of assay buffer, 10 µL of Hemin, and 40 µL of boiled sample; sample wells received 120 µL of assay buffer, 10 µL of hemin, and 40 µL of sample; COX-1 or 2 specific inhibition wells received 110 µL of assay buffer, 10 µL of hemin, 40 µL of sample, and 10 µL of either DuP-697 (5-(4-Chlorophenyl)-1-(4-methoxyphenyl)-3-trifluoromethylpyrazole) or SC-560 (5-(4-Chlorophenyl)-1-(4-methoxyphenyl)-3-(trifluoromethyl)-1H-pyrazole). The plate was gently shaken and incubated for five minutes at 25 °C, after which 20 µL of colorimetric substrate was added to each well. The reaction was initiated by adding 20 µL of arachidonic acid solution, followed by a five-minute incubation at 25 °C. Absorbance was measured at 590 nm, using a microplate reader.

Data analysis involved calculating the average absorbance for each set of replicates and subtracting the background values from the corresponding sample and inhibitor wells. The total COX activity was determined according to the kit’s formula, accounting for the requirement of two TMPD (N,N,N′,N′-Tetramethyl-p-phenylenediamine) molecules to reduce PGG2 (Prostaglandin G_2_) to PGH2 (Prostaglandin H_2_). The percentage inhibition by each inhibitor was calculated to determine the relative proportions of COX-1 and COX-2 activity in the samples.

### 2.11. Data Processing and Statistical Analysis

All the extractions and quantifications were conducted in triplicate to ensure reproducibility and statistical reliability. Final values were expressed as the mean ± standard deviation (SD) from three independent measurements per treatment. A one-way ANOVA was performed to evaluate the differences among the groups. When the ANOVA indicated statistical significance (*p* < 0.05), Tukey’s Honestly Significant Difference (HSD) post hoc test was applied to identify pairwise differences. All the statistical analyses were conducted using the JASP software (version 0.19.3) [JASP Team, University of Amsterdam, Amsterdam, The Netherlands].

## 3. Results

**Figure 1:**
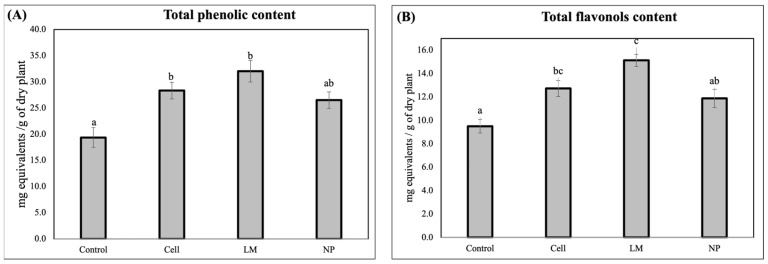
(**A**) Total phenols (mg of gallic acid equivalent per gram of dry extract) and (**B**) total flavonols (mg of catechin equivalent per gram of dry extract). Values correspond to the mean ± SD. Different letters (a, b, c) indicate statistically significant differences among treatments as determined by a one-way ANOVA, followed by Tukey’s HSD post hoc test (*p* < 0.05).

The quantification of the total phenolic content revealed that all the treatments significantly decreased the total phenols and total flavonols in the rosemary extracts (Figure 1).

**Figure 2:**
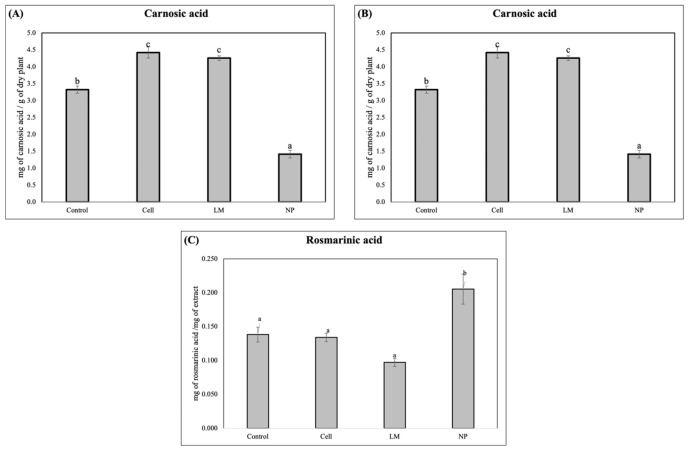
The total content of (**A**) carnosic acid, (**B**) carnosol, and (**C**) rosmarinic acid in rosemary plants after treatment are expressed as mg of active compound per mg of dry extract. Values correspond to the mean ± SD. Different letters (a, b, c) indicate statistically significant differences among treatments as determined by a one-way ANOVA, followed by Tukey’s HSD post hoc test (*p* < 0.05).

The HPLC analysis revealed a significantly lower concentration of the diterpene, carnosol, in the LM and NP, while a significant increase in rosmarinic acid was detected in the NP treatments (Figure 2).

The HPLC–MS analysis in scan mode of the extracts revealed distinct variations in the metabolic profiles, specifically in the relative abundance of secondary metabolites among the control, cell-treated, LM-treated, and NP-treated plant extracts (Table 1) (Figure S2 in the Supplementary Materials). Notably, the NP-treated samples exhibited selective upregulation of several key compounds, particularly within the phenylpropanoid and flavonoid classes. For instance, rosmarinic acid and caffeic acid among the phenylpropanoid class, luteolin derivatives among the flavonoid class, carnosol in the diterpene class, and all of the triterpenes, except for asiatic acid. These changes suggest the targeted activation of specific branches of secondary metabolism in response to nanoparticle treatment. Conversely, other compounds such as gallocatechin or syringic acid were downregulated, indicating a possible metabolic reallocation or competitive flux through biosynthetic pathways. The detailed distribution of relative changes in metabolite abundance is summarized in Table 1.

The antioxidant capacity (AC) of the extracts, according to the DPPH Assay, is presented in Table 2. The IC_50_ values (expressed in µg/mL) indicated similar antioxidant activity across most of the treatments when compared to the control, suggesting that the overall free radical scavenging capacity remained largely unaffected. Specifically, the cells and LM extracts exhibited slightly higher IC_50_ values (58.00 and 54.53 µg/mL, respectively), which correspond to a modest reduction in antioxidant potency relative to the control. In contrast, the NP treatment demonstrated an IC_50_ value of 52.64 µg/mL, closely comparable to that of the control (52.81 µg/mL), indicating that the nanoparticle application maintained the antioxidant efficacy. These subtle variations may reflect differences in the qualitative composition of the antioxidant compounds rather than total antioxidant capacity, highlighting the need for further analysis of specific metabolite contributions to the observed activity.

**Figure 3:**
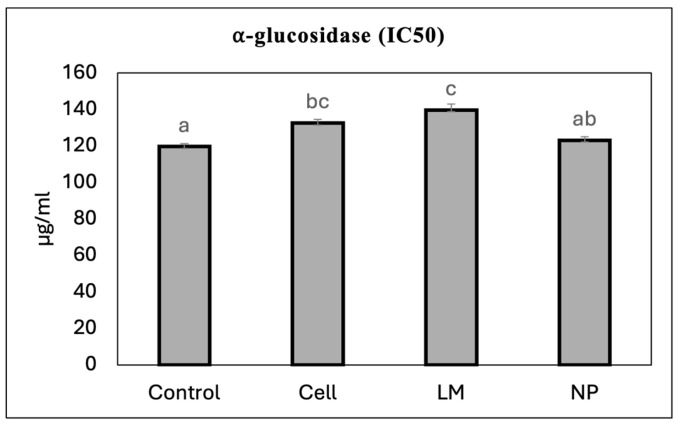
IC_50_ values (µg of extract/mL) for alpha-glucosidase inhibition of *Salvia rosmarinus* extracts. Values correspond to the mean ± SD. Different letters (a, b, c) indicate statistically significant differences among treatments as determined by a one-way ANOVA, followed by Tukey’s HSD post hoc test (*p* < 0.05).

The extracts’ potential to inhibit α-glucosidase activity was calculated as the IC_50_ value (Figure 3). The cells and LM extracts increased the IC_50_ value as compared to the controls, therefore achieving worse results than the controls and NPs.

**Figure 4:**
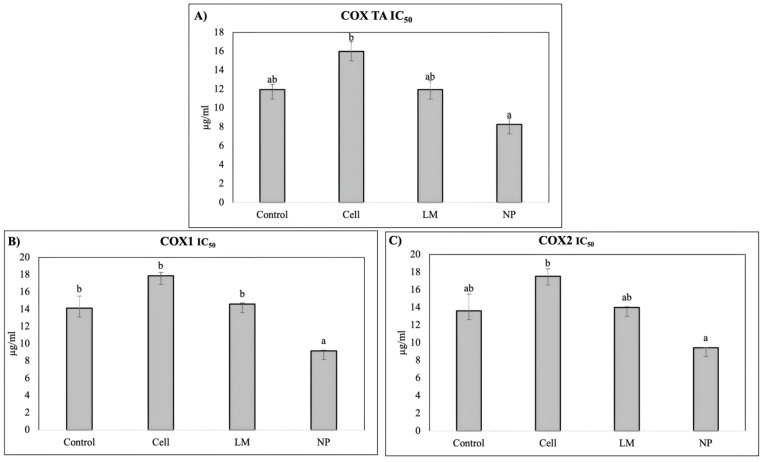
IC_50_ values (µg of extract/mL) for (**A**) total COX; (**B**) COX-1; and (**C**) COX-2 inhibition of *Salvia rosmarinus* extracts. Values correspond to the mean ± SD. Different letters (a, b) indicate statistically significant differences among treatments as determined by a one-way ANOVA, followed by Tukey’s HSD post hoc test (*p* < 0.05).

The extract’s anti-inflammatory potential was evaluated based on the ability to inhibit cyclooxygenase (COX), evaluating COX-1, COX-2, and total COX (Figure 4).

The COX inhibition assays revealed a significant decrease in the IC_50_ values for rosemary extracts derived from NP-treated plants, indicating enhanced inhibitory potency against both cyclooxygenase isoforms, COX-1 and COX-2. This suggests that nanoparticle treatment effectively increased the concentration or bioavailability of active compounds capable of modulating inflammatory pathways. The improved inhibition of COX enzymes highlights the potential of NP-mediated metabolic induction to augment the anti-inflammatory properties of rosemary extracts.

## 4. Discussion

The ability of AgNPs coated with beneficial bacteria metabolites to trigger *Salvia rosmarinus* metabolism during the postharvest period has been shown to be a valuable biotechnological tool to improve the anti-inflammatory potential of rosemary extracts. AgNPs cause a different response to that of the original strain (cells) or the complex mixture of bacterial metabolites (LM), showing that the effects are attributed to the formulation of metabolites at the nanoscale, which enables deeper penetration and a concomitant more intense response. Despite the fact that the described effects are limited to rosemary in the present study, a wider effect on other plant species is anticipated as Pseudomonas strains are able to trigger many plant species [25]; more precisely, *Pseudomonas shirazensis* NFV3 is able to enhance plant growth and metabolism in different plant species like Arabidopsis [31], as well as tomato [32] and berries [18,25]

The HPLC and HPLC–MS analyses conducted in this study revealed a significant metabolic modification in the samples treated with AgNPs, demonstrated by the increased accumulation of rosmarinic acid and a decrease in carnosol (Figure 2). These metabolites are well-documented for their potent antioxidant and anti-inflammatory properties, contributing substantially to the pharmacological value of *Salvia rosmarinus* extracts [5,6,7]. The observed modification of these compounds indicates the robust activation of phenylpropanoids and the modification of diterpenoid biosynthetic pathways, which are typically involved in the plant’s response to biotic and abiotic stimuli [10,12].

Interestingly, the NP treatment resulted in a markedly lower concentration of carnosol and carnosic acid compared to the control and the other treatments. This suggests the occurrence of a selective modulation or metabolic reallocation triggered by the nanoparticle formulation, potentially involving the differential regulation of key enzymes, such as geranylgeranyl diphosphate reductase or ferruginol synthase, which are crucial in the diterpenoid biosynthetic cascade [33]. Selective metabolic reprogramming in response to nanoparticles has been previously described in species such as *Arabidopsis* thaliana and *Medicago sativa*, wherein metallic and polymeric nanoparticles induced divergent transcriptomic responses and metabolite fluxes depending on the particle size, composition, and surface functionalization [20,21]. Such selective responses may be mediated by a combination of oxidative signaling, hormone crosstalk, and epigenetic reconfiguration, emphasizing the complexity of nanoparticle–plant interactions [24].

The dual effect observed, wherein some branches of the secondary metabolism are enhanced while others are downregulated, underscores the specificity and potential of NP-based elicitation to fine-tune metabolite profiles. This complexity highlights the necessity of integrating omics approaches (transcriptomics, proteomics, and metabolomics) to comprehensively elucidate the molecular mechanisms underpinning such metabolic plasticity [21,23]. Moreover, studies involving nanoparticle uptake, translocation, and subcellular localization would further clarify how these materials influence cellular metabolism at the biochemical level [20].

The HPLC–MS profiling further supported these findings, showing that all the rosemary extracts exhibited a similar profile, and all showed the same composition, despite the profiles from NP-treated plants showing increased relative peak areas compared to the controls (Table 1); interestingly, triterpenes were noticeably increased, specially betulinic and ursolic acid. This broad-spectrum metabolic activation implies that nanoparticle exposure may act as a general elicitor, potentially triggering multiple biosynthetic pathways through stress-induced signaling networks, such as ROS bursts, MAPK cascades, or jasmonate signaling [21,24]. These networks are known to orchestrate the transcriptional reprogramming of specialized metabolism genes, contributing to the activation of complex metabolite assemblages that confer adaptive advantages to the plant, while simultaneously increasing extract bioactivity [5].

In pharmacological terms, this compositional remodeling translated into functional improvements in terms of anti-inflammatory potential, but not in regard to the hypoglycemic effects. Notably, the COX inhibition assays demonstrated a significant decrease in the IC_50_ values for the extracts derived from NP-treated plants, indicating enhanced inhibitory activity against both COX-1 and COX-2 isoforms. This increased potency correlates with the observed accumulation of specific anti-inflammatory compounds, such as rosmarinic acid, diosmetin, micromeric acid, and carnosol, all of which are known to interfere with eicosanoid synthesis or modulate NF-κB signaling [5,7,14]. This pharmacological enhancement occurred despite the absence of significant differences in the total phenolic content, emphasizing that it is the qualitative remodeling of the bioactive profile, not merely a quantitative increase, that defines extract potency and activity [10,23].

These findings indicate that nanoparticle treatment not only expands the overall metabolite pool, but also specifically modulates the composition and functional direction of the secondary metabolome to enhance its anti-inflammatory potential. This insight is particularly valuable for phytopharmaceutical applications, wherein the therapeutic efficacy of plant extracts often depends on the presence of synergistic combinations of bioactive compounds rather than high concentrations of a single molecule [5,9,34]. In this context, NP-mediated elicitation emerges as a promising tool in the field of plant metabolic engineering, enabling the controlled enhancement of targeted pharmacological properties [21,24].

In contrast to the highly specific metabolic remodeling observed in the NP-treated plants, the total phenolic quantification showed no significant differences between the NP and the control, suggesting that the overall pool of phenolic compounds remained relatively stable. However, both cells and LM treatments led to a statistically significant increase in the total phenolic content, reflecting a broader, non-selective stimulation of phenylpropanoid metabolism [6,12]. This observation indicates that while classical biotechnological tools, such as plant cell cultures or metabolic liquids, can upregulate entire pathways, nanoparticle formulations offer the advantage of selective pathway modulation [21,23].

The marked increase in flavonol content observed in the extracts from the LM-treated plants further supports this differential mode of action. It is plausible that LM contains endogenous or exogenously induced elicitors, such as signaling peptides or microbial metabolites, capable of activating transcription factors (e.g., MYB12, TTG1) involved in flavonoid biosynthesis [20,24] that are not able to reduce silver and, therefore, are not present in the organic crown of NP. Given the central role of flavonoids in oxidative stress protection, UV shielding, and hormone transport, such increases have physiological relevance beyond their pharmacological interest and may improve plant resilience and postharvest extract quality when delivered along the plant cycle [9,10].

Regarding antioxidant activity, the IC_50_ values from the DPPH assays showed only modest variations across the treatments, with the NP extracts exhibiting nearly identical antioxidant potency to the control samples. Although the cells and LM treatments yielded slightly higher IC_50_ values, suggesting marginally lower scavenging capacity, these changes are unlikely to be biologically significant. Rather, they likely reflect qualitative shifts in the antioxidant profile, wherein metabolites with high radical scavenging potential may be replaced by others with lower activity but greater relevance to anti-inflammatory or cytoprotective functions [5,7,10]. This further illustrates that antioxidant capacity is a multifactorial trait that is not governed solely by its phenolic abundance, but by the chemical nature, redox potential, and synergistic interactions of the constituent metabolites.

## 5. Conclusions

This study demonstrates that formulating *Pseudomonas shirazensis* bacterial metabolites in nanoparticles can effectively modulate the secondary metabolism of *Salvia rosmarinus*, leading to significant alterations in the metabolite composition and pharmacological activity, even when delivered during the postharvest period. Nanoparticle treatments offer a unique advantage by enabling targeted remodeling of metabolite profiles, enhancing specific anti-inflammatory compounds without indiscriminately increasing the total phenolic content. These findings open up new avenues for the application of nanotechnology in regard to phytopharmaceutical optimization and targeted metabolic engineering. Further work integrating transcriptomic and proteomic data, as well as in vivo pharmacological and toxicological assessments, will be essential to fully exploit the therapeutic potential of nanoparticle-enhanced medicinal plant extracts.

In summary, delivering NP to *Salvia rosmarinus* during the postharvest period represents a promising tool to further increase natural bioactives, improving their anti-inflammatory, antihyperglycemic, and antioxidant properties. This tool appears to be an excellent alternative for the plant extracts industry. However, further research is required to overcome current challenges related to standardization, mechanistic elucidation, and clinical efficacy.

## 6. Patents

A patent application has been filed with reference P202430961, entitled “*Pseudomonas shirazensis* NVF3 y sus metabolitos vehiculizados en nanopartículas de plata como promotores del crecimiento vegetal, estimulantes de la adaptación en situaciones de estrés hídrico y del metabolismo secundario de interés farmacológico y alimentario”. The IET+OE has been published and recognizes patentability. It is not public yet.

## Figures and Tables

**Table 1:** Relative (%) area of the different secondary metabolites identified in HPLC–MS in scan mode for control, cells, LM, and AgNP treatments. Metabolites with relevant increases in relative areas in response to the NP treatment are highlighted in blue.

	Compound	CONTROL	CELLS	LM	NP
S	Rosmarinic acid	7.87	6.69	7.65	10.72
S	Caffeic acid	2.76	3.01	2.83	4.23
S	Quinic acid	9.49	10.88	12.19	9.43
S	Syringic acid	1.19	1.32	1.16	0.77
S	Gallocatechin	12.85	15.10	13.41	5.44
	**TOTAL PHENILPROPANOIDS**	**34.16**	**36.99**	**37.24**	**30.59**
F	Luteolin	0.37	0.45	0.33	0.38
F	Hesperidin	0.45	0.37	0.32	0.52
F	6-Hydroxyluteolin-7-O-glucoside	0.59	0.59	0.46	0.54
F	Luteolin 7-O-rutinoside	0.32	0.32	0.25	0.33
F	Isorhamnetin-3-O-glucoside	2.92	2.66	2.66	3.64
F	Apigenin-7-O-glucoside	0.18	0.17	0.18	0.16
F	Isorhamnetin-3-O-rutinoside	0.09	0.08	0.08	0.10
F	Diosmin	0.40	0.48	0.41	0.55
F	Hispidulin-7-O-glucoside o Luteolin-7-O-	1.87	1.82	1.88	2.47
F	Isorhamnetin	0.42	0.56	0.39	0.45
F	Luteolin 3′-acetyl-O-glucuronide II	0.37	0.41	0.31	0.54
F	Diosmetin	0.57	0.78	0.67	0.76
F	Pectolinarigenin	1.57	2.10	1.76	1.55
F	Genkwanin	0.21	0.24	0.12	0.28
	**TOTAL FLAVONOIDS**	**10.33**	**11.03**	**9.83**	**12.27**
D	**Carnosic acid**	18.73	15.90	15.94	13.53
D	**Rosmanol isomer**	18.45	15.70	17.78	2.87
D	Carnosol	3.22	3.43	2.60	22.38
D	Rosmanol	7.33	8.43	7.94	8.28
D	Rosmadial	1.28	1.98	2.36	1.91
D	12-methoxy-carnosic acid	2.45	2.56	2.86	3.32
	**TOTAL DITERPENS**	**51.46**	**48.00**	**49.48**	**52.30**
T	Asiatic acid	0.91	0.85	0.62	0.82
T	Corosolic acid	0.94	0.83	0.79	1.04
T	Micromeric acid	0.56	0.61	0.47	0.85
T	Betulinic acid	0.76	0.76	0.62	0.99
T	Ursolic acid	0.89	0.93	0.96	1.15
	**TOTAL TRITERPENS**	**4.05**	**3.98**	**3.45**	**4.84**

**Table 2:** IC_50_ values of control, cells, LM, and NP extracts and positive controls in regard to the DPPH antioxidant assay. CL corresponds to confidence limits.

Treatment or Positive Control	IC_50_ (µg/mL)	CL (µg/mL)
Control	52.81	50.10–55.74
Cell	58.00	55.23–61.09
LM	54.53	50.58–58.81
NP	52.64	45.48–54.96
Gallic acid	0.96	8.54 × 10^−7^–1.07 × 10^−6^
Rosmarinic acid	5.14	5.04 × 10^−6^–5.28 × 10^−6^

## Data Availability

The data that support the findings of this study are stored on the computers at CEU San Pablo University and are available from the corresponding author upon reasonable request.

## Supplementary Materials

The following supporting information can be downloaded at: https://www.mdpi.com/article/10.3390/antiox14080931/s1, Figure S1. Supplementary Material. Calibration curves for quantification of phenolic compounds Using HPLC. (A) Rosmarinic acid calibration curve. (B) Carnosic acid calibration curve. (C) Carnosol calibration curve. Figure S2. Supplementary Material. HPLC–MS chromatogram of NP extracts.

## Abbreviations

The following abbreviations are used in this manuscript:

Cell	*Pseudomonas shirazensis* NFV3 cells suspended in water
LM	Liquid medium containing *Pseudomonas shirazensis* NFV3 metabolites
NP	*Pseudomonas shirazensis* NFV3 metabolites (LM) formulated in silver nanoparticles
