# Supplementary material for: Enhanced Anti-Inflammatory Effects of Rosemary (Salvia rosmarinus) Extracts Modified with Pseudomonas shirazensis Nanoparticles
Source: Antioxidants (Basel). 2025 Jul 29;14(8):931. doi: 10.3390/antiox14080931 (PMC12383122; doi:10.3390/antiox14080931)
Supplement: Supplementary file 1 [file antioxidants-14-00931-s001.zip › antioxidants-3750266-supplementary.pdf]

Supplementary Materials

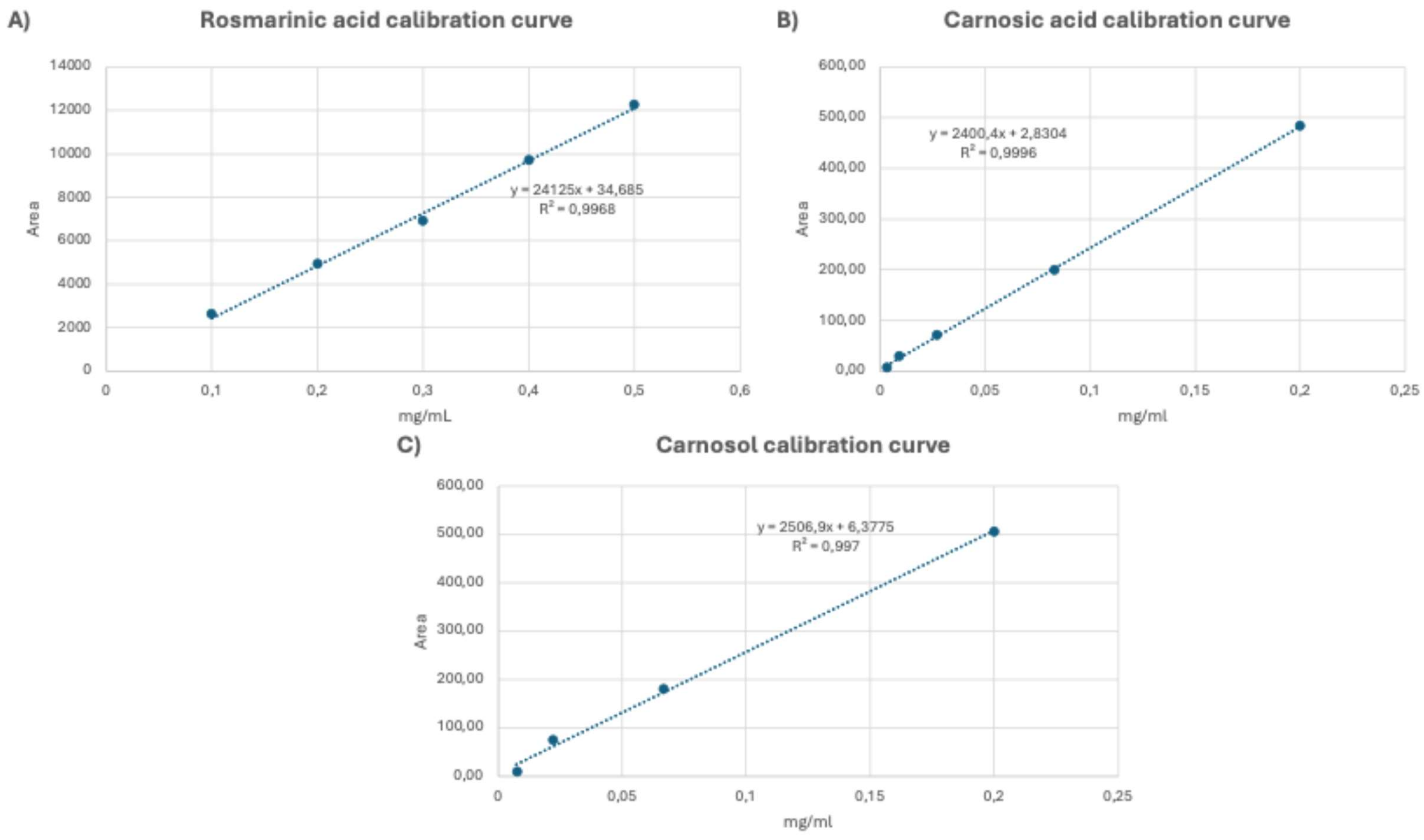

Figure S1. Calibration Curves for Quantification of Phenolic Compounds by HPLC. A) Rosmarinic acid calibration curve. B) Carnosic acid calibration curve. C) Carnosol calibration curve.

File: d:\data\muestras 2025\marzo\lctq250245_01_27740.d\lctq250245_01_27740.d.xms
Sample: LCTQ250245 Operator:
Scan Range: 1 - 2940 Time Range: 0.04 - 20.00 min. Date: 3/14/2025 1:00 PM
Sample Notes: 02195. PP-NPN

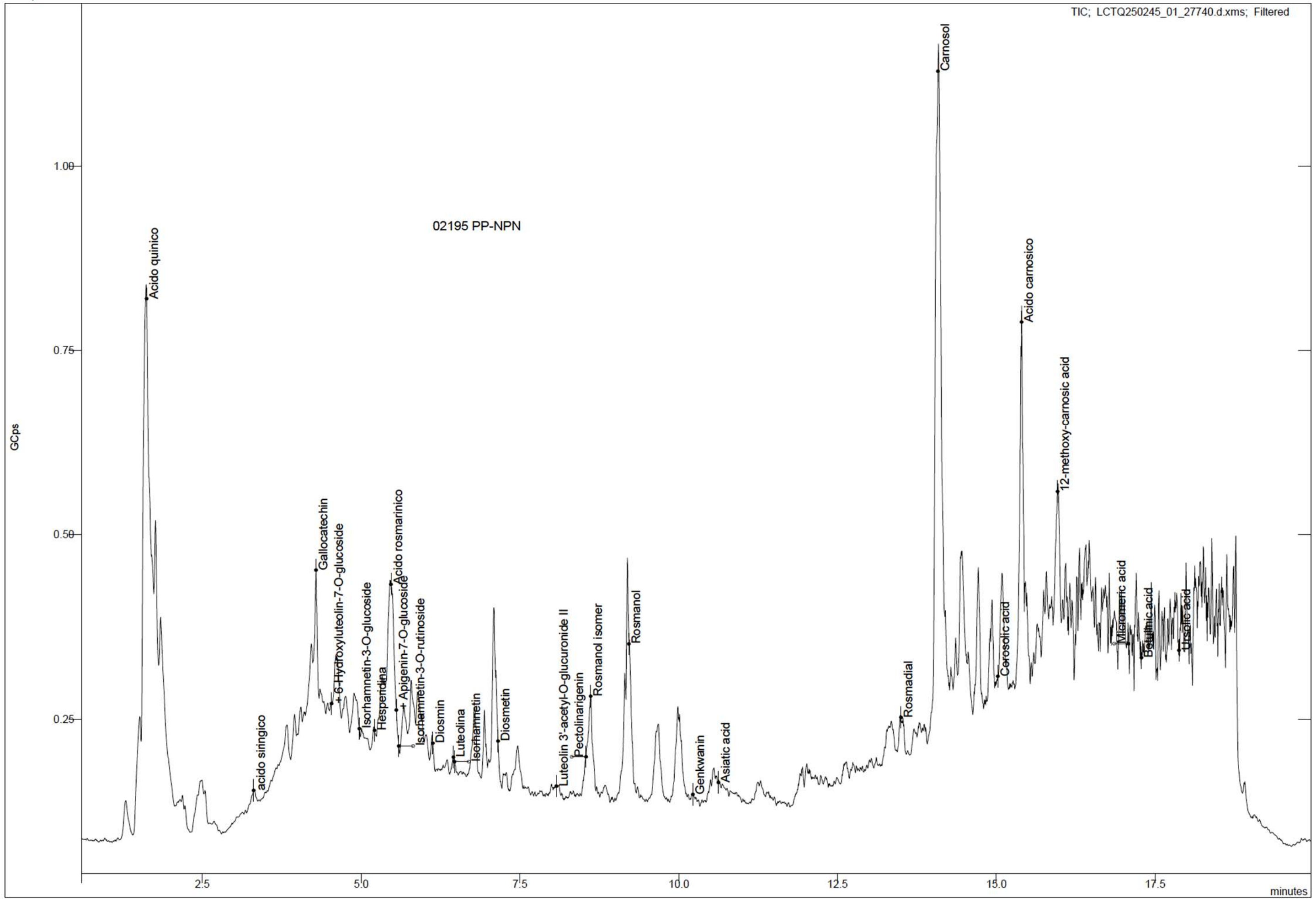

Figure S2. HPLC-MS chromatogram of NP extracts.
